# Assessment of Clinical Significance of Molecular *Streptococcus agalactiae* Detection in Patients With Suspected Pneumonia

**DOI:** 10.1093/ofid/ofaf781

**Published:** 2025-12-24

**Authors:** Mona Mustafa Hellou, Guyu Li, Rita Igwilo-Alaneme, Abinash Virk, Elias Hellou, Zane Lancaster, Robin Patel

**Affiliations:** Division of Clinical Microbiology, Department of Laboratory Medicine and Pathology, Mayo Clinic, Rochester, Minnesota, USA; Division of Pediatric Infectious Diseases, Department of Pediatrics, Mayo Clinic, Rochester, Minnesota, USA; Division of Public Health, Infectious Diseases and Occupational Medicine, Department of Medicine, Mayo Clinic, Rochester, Minnesota, USA; Division of Public Health, Infectious Diseases and Occupational Medicine, Department of Medicine, Mayo Clinic, Rochester, Minnesota, USA; Department of Cardiovascular Medicine, Mayo Clinic, Rochester, Minnesota, USA; Division of Clinical Microbiology, Department of Laboratory Medicine and Pathology, Mayo Clinic, Rochester, Minnesota, USA; Division of Clinical Microbiology, Department of Laboratory Medicine and Pathology, Mayo Clinic, Rochester, Minnesota, USA; Division of Public Health, Infectious Diseases and Occupational Medicine, Department of Medicine, Mayo Clinic, Rochester, Minnesota, USA

**Keywords:** colonization, group B *Streptococcus*, multiplex polymerase chain reaction, pneumonia, *Streptococcus agalactiae*

## Abstract

**Background:**

*Streptococcus agalactiae* (group B *Streptococcus* [GBS]) may cause pneumonia or may colonize the respiratory tract, making its clinical significance uncertain when detected in respiratory specimens. This study aimed to assess whether clinicians interpreted GBS detected by the BIOFIRE Pneumonia Panel (BF-PP) as a pathogen implicated in pneumonia.

**Methods:**

This retrospective cohort study included adult patients hospitalized with suspected pneumonia at Mayo Clinic, Rochester, between September 2020 and February 2025. Cases were independently classified into pathogen and nonpathogen groups by 2 infectious diseases (ID) specialists, with a third reviewer resolving disagreements. Clinical characteristics and outcomes of both groups were recorded. A subgroup analysis of patients who had an ID consultation was performed.

**Results:**

A total of 109 cases were included. GBS was considered a pneumonia pathogen in 47.7% of cases and a nonpathogen in 52.3%. ID consultation was performed in 33.0% of cases, with GBS considered a pathogen in 30.6% of those. Common comorbid conditions included pulmonary, gastrointestinal, neurologic, and cardiovascular disease and obesity. Rates of endotracheal intubation were similar in the pathogen and nonpathogen groups (51.9% vs 50.9%, respectively), with the in-hospital mortality rate being numerically but not significantly higher in the former versus the latter (21.2% vs 14.0%; *P* = .33); findings were similar in the ID-assessed subgroup. In 76.1% of GBS detections, other microorganisms—most commonly *Staphylococcus aureus*—were codetected. Only 10.2% of BF-PP GBS-positive specimens were culture positive for GBS.

**Conclusions:**

While GBS is not an uncommon pneumonia pathogen, the clinical significance of its detection by BF-PP is uncertain in many cases.

## Teaser

In more than half of BIOFIRE Pneumonia Panel *Streptococcus agalactiae* detections, the organism was considered nonpathogenic. Assessing clinical relevance can be challenging, highlighting the need for means to distinguish a true pneumonia pathogen from respiratory tract colonization.

*Streptococcus agalactiae* (group B *Streptococcus* [GBS]) is a human pathogen primarily associated with invasive neonatal and puerperal infections [1, 2]. While its incidence in these groups has declined due to screening and, for high-risk cases, antibiotic prophylaxis, over the last decades it has been increasingly reported as a cause of infection in the nonpregnant adult population [3–6].

Clinical manifestations of invasive GBS infections in nonpregnant adults are diverse, including bacteremia without an identified focus, skin and soft-tissue infection, pneumonia, osteoarticular infection, and urinary tract infection, with meningitis and endocarditis occurring less frequently [7–11]. GBS is an uncommon cause of pneumonia; however, pneumonia as a manifestation of invasive GBS disease has been reported in 6%–25% of cases [12–14] and is considered a severe infection with a high mortality rate [15]. Although invasive GBS disease may occur in otherwise healthy adults, most patients have underlying clinical conditions [16], including diabetes mellitus, cancer, cirrhosis or neurologic disorders, the last increasing the risk of aspiration [17]. However, the role of GBS as the sole determinant of infection severity remains unclear, as many severe infections are polymicrobial, often involving *Staphylococcus aureus*.

The BIOFIRE Pneumonia Panel (BF-PP) is a multiplex molecular assay that can detect multiple microorganisms implicated in pneumonia from respiratory specimens [18]. Included in its targets is GBS. With increasing use of this panel in clinical practice, it is expected that there will be increased detection of GBS in respiratory specimens. However, since GBS can be a colonizer of the upper respiratory tract [19], it may be unclear whether its detection represents respiratory tract colonization or GBS pneumonia.

In the current study, we investigated whether clinicians consider GBS detected by the BF-PP a pathogen implicated in pneumonia. We also compared clinical characteristics and outcomes in those in whom BF-PP–detected GBS was classified as a pathogen versus a nonpathogen.

## METHODS

This retrospective cohort study was conducted at Mayo Clinic in Rochester, Minnesota. Data were collected from 2 cohorts, both including adult patients (≥18 years old) either hospitalized with suspected pneumonia or in whom pneumonia was suspected during their hospitalization and who had a positive BF-PP result for GBS. The first cohort consisted of patients enrolled in a randomized controlled trial conducted at Mayo Clinic between 15 September 2020 and 18 September 2022 evaluating the utility of the BF-PP compared with standard microbiological testing in guiding antibiotic use [20]. The second cohort included patients hospitalized between 19 September 2022 and 28 February 2025. Patients were excluded if they were discharged directly from the emergency department without further interpretation of a BF-PP result, or if their electronic medical record (EMR) data were incomplete or unavailable, precluding assessment of study outcomes. For those with 2 specimens collected during the same hospitalization, only the first was included.

In the first cohort, diagnosis of pneumonia was defined based on clinical and radiographic findings as assessed by an antimicrobial stewardship team or the infectious diseases (ID) consultant managing the patient. In the second cohort, the presence or absence of pneumonia was determined based on documentation in the EMR. Pneumonia was classified as community-acquired pneumonia (CAP) if acquired in the community, as hospital-acquired pneumonia (HAP) if it developed ≥48 hours after admission, as healthcare-associated pneumonia (HCAP) if contracted outside the hospital but in context of recent healthcare exposure, and as ventilator-associated pneumonia (VAP) if it developed after ≥48 hours of intubation. Patients initially (ie, at the time of BF-PP testing) considered to possibly have pneumonia but ultimately determined not to have pneumonia were included.

### Approval and Consent

The Mayo Clinic Institutional Review Board approved the study protocol. A waiver of informed consent was granted, in accordance with 45 CFR §46.116, and a waiver of Health Insurance Portability and Accountability Act authorization, in accordance with applicable regulations.

### Data Collection

Patient demographics, underlying comorbid conditions, laboratory findings, antibiotic treatment administered before BF-PP testing and in-hospital mortality during the index hospitalization were retrospectively extracted from the EMR. The interpretation of GBS detection by the BF-PP was based on the documented assessment by the treating clinician (non-ID clinician) and, when available, ID specialist, regarding the role of GBS in pneumonia.

Each case was classified into 1 of 2 groups, the pathogen group, in which GBS was interpreted as a pathogen implicated in pneumonia, and the nonpathogen group, in which GBS was considered a colonizer of the respiratory tract or of unclear clinical significance. These interpretations were independently reviewed and categorized by 2 ID clinicians. In cases of disagreement, a third reviewer adjudicated to reach a final classification. Cases were classified into the pathogen group if any of the following criteria was met: documentation of “GBS pneumonia” or “*Streptococcus agalactiae* pneumonia” in the discharge diagnosis or clinician notes; clinical documentation indicating that GBS was considered a causative agent of pneumonia, either alone or with other microorganisms; or detection of GBS as the sole bacterial pathogen by the BF-PP, followed by targeted antibiotic therapy consistent with treatment of GBS pneumonia.

Pulmonary, gastrointestinal (GI), neurologic, cardiovascular, and renal comorbid conditions were recorded. Pulmonary comorbid conditions included asthma, chronic obstructive pulmonary disease, bronchiectasis, interstitial lung disease, lung cancer, and ventilator dependence. GI diseases included esophagitis, gastroesophageal reflux disease, gastroparesis, chronic pancreatitis, cholecystitis, cirrhosis, hepatitis C infection, GI cancer, and past surgical interventions involving the GI tract. Neurologic comorbid conditions included seizure disorder, cognitive disorder, Parkinson disease, neuropathy, and history of cerebrovascular accident with neurologic sequalae. Cardiovascular comorbid conditions included hypertension, rhythm disorder, cardiomyopathy, coronary artery disease, and valvular disease. Renal diseases included chronic kidney disease, renal amyloidosis, and nephrolithiasis.

### Specimens

Respiratory specimens included sputum, tracheal secretion, and bronchoalveolar lavage (BAL) fluid specimens. Each specimen was analyzed using the BF-PP and conventional culture collected at the same time. No specimen processing was performed before testing with the BF-PP. For cultures, specimens were inoculated onto blood, chocolate and eosin methylene blue agar (Becton Dickinson) and incubated at 35°C for 2 days, with the initial reading performed after 16–24 hours. For sputum specimens, only those containing 25 or fewer squamous epithelial cells per low-power field on Gram stain were considered acceptable and processed for both culture and BF-PP testing. Testing on specimens with >25 squamous epithelial cells per low-power field was canceled [21]. GBS detections by the BF-PP were reported semiquantitatively, as 10^4^, 10^5^, 10^6^ or ≥10^7^ genomic copies/mL.

### Statistical Analysis

Data were analyzed using R software, version 4.5.0 (R Foundation for Statistical Computing) within the RStudio integrated development environment (RStudio). Patient characteristics were summarized using descriptive statistics. Continuous variables were reported as medians with interquartile ranges (IQRs) and categorical variables as frequencies and percentages. Comparisons between categorial variables were made using χ^2^ or Fisher exact tests, as appropriate. Continuous variables were compared using the Mann-Whitney *U* test. A 2-sided significance level of α = .05 was used for statistical tests.

## RESULTS

**Figure 1.**
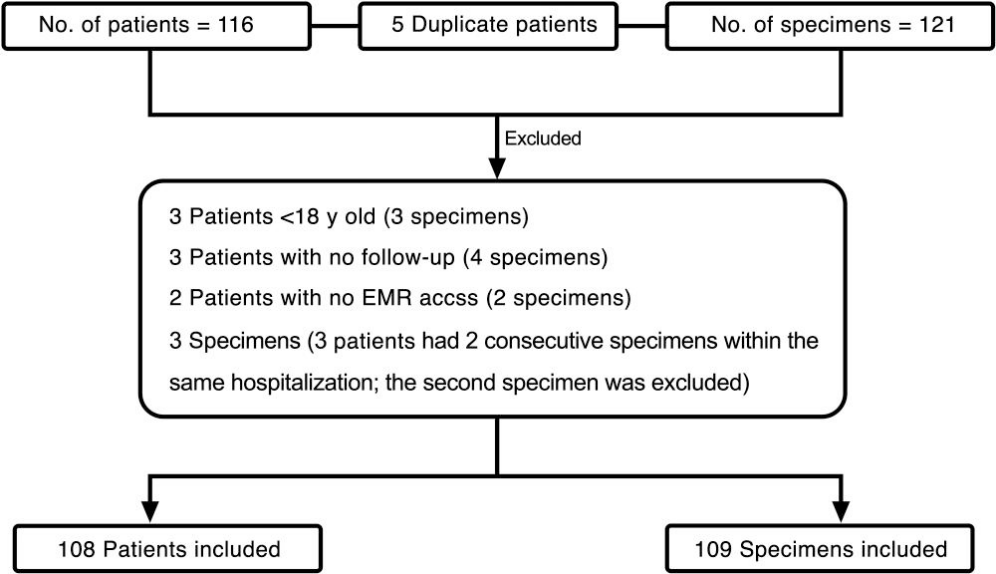
Study outline. Abbreviation: EMR, electronic medical record.

During the study period, 116 patients tested positive for GBS by the BF-PP, yielding a total of 121 respiratory specimens (5 patients had 2 specimens tested). Twelve specimens from 8 patients were excluded for the following reasons: 3 patients (3 specimens) were <18 years of age, 3 (4 specimens) had no follow-up; and 2 (2 specimens) did not provide permission for access to their medical record. In addition, for 3 patients from whom 2 specimens were collected during the same hospitalization, the second specimen was excluded. One patient had 2 specimens, each collected during separate hospitalizations; each was considered an independent index event for a new episode of suspected pneumonia and therefore both were included. In total, 109 specimens were included in the final analysis from 108 patients (Figure 1).

Respiratory specimens included 57 sputum, 42 tracheal secretion, and 10 BAL fluid specimens. Of these, 75.2% (82 of 109) were from patients diagnosed with pneumonia. Among the pneumonia-associated cases, 69.5% (57 of 82) were classified as CAP, 12.2% (10 of 82) as HAP, 9.8% (8 of 82) as HCAP, and 8.5% (7 of 82) as VAP.

### Patient Data

Among the 109 cases, GBS was considered by non-ID clinicians as a pathogen implicated in pneumonia in 47.7% of cases (52 of 109) (pathogen group), while in 52.3% (57 of 109) it was classified as a colonizer of the respiratory tract or of unclear clinical significance (nonpathogen group). ID consultation was performed in 33.0% of cases (36 of 109). Among these, GBS was classified as a pathogen in 30.6% (11 of 36) and a colonizer or of unclear clinical significance in 69.4% (25 of 36).

Table 1 presents a comparison of demographics, clinical and laboratory findings between the pathogen and nonpathogen groups, as classified by non-ID clinicians. The median age (IQR) was comparable between the pathogen and nonpathogen groups, 59 (49–72) years and 61 (44–68) years, respectively (*P* = .58). Both groups showed a male predominance, with male patients comprising 63.5% in the pathogen group and 66.7% in the nonpathogen group (*P* = .73). Pulmonary comorbid conditions were present in 53.8% of those in the pathogen group and 43.9% in the nonpathogen group (*P* = .30). GI comorbid conditions were reported in 53.8% of the pathogen and 59.6% of the nonpathogen group (*P* = .54). Neurologic disease was present in 42.3% and 49.1%, respectively (*P* = .48). Cardiovascular diseases were observed in 67.3% in the pathogen and 84.2% in the nonpathogen group (*P* = .04), obesity in 50.0% and 56.1%, respectively (*P* = .52), and renal disease in 23.1% and 22.8% (*P* = .97). Cancer was reported in 15.4% in the pathogen group and in 24.6% in the nonpathogen group (*P* = .23).

**Table 1.** Baseline Characteristics and Clinical Outcomes in the *Streptococcus agalactiae* Pathogen and Nonpathogen Assignment Groups According to Non–Infectious Diseases Clinician Assessments

Characteristic	Patients, No. (%)^a^		*P* Value
	Pathogen Group (n = 52)	Nonpathogen Group (n = 57)	
Age, median (IQR), y	59 (49–72)	61 (44–68)	.58
Male sex	33 (63.5)	38 (66.7)	.73
Pulmonary disease	28 (53.8)	25 (43.9)	.30
Gastrointestinal disease	28 (53.8)	34 (59.6)	.54
Neurologic disease	22 (42.3)	28 (49.1)	.48
Cardiovascular disease	35 (67.3)	48 (84.2)	.04
Renal disease	12 (23.1)	13 (22.8)	.97
Diabetes	20 (38.5)	25 (43.9)	.57
Obesity	26 (50.0)	32 (56.1)	.52
Cancer	8 (15.4)	14 (24.6)	.23
Smoking	12 (23.1)	12 (21.1)	.80
Substance use (including alcohol)	10 (19.2)	18 (31.6)	.14
Time from admission to BF-PP testing, median (IQR), d	1 (0–2)	1 (0–4)	.20
Intubated during hospitalization	27 (51.9)	29 (50.9)	.91
In-hospital death	11 (21.2)	8 (14.0)	.33
Pneumonia	43 (82.7)	39 (68.4)	.08
Community-acquired pneumonia	32 (61.5)	25 (43.9)	.06
Prior antibiotic therapy	42 (80.8)	42 (73.7)	.38
Time to antibiotic initiation			
<24 h	27 (64.3)	21 (50.0)	.19
24–48 h	7 (16.7)	9 (21.4)	.58
>48 h	8 (19.0)	12 (28.6)	.31
Leukocyte count, median (IQR), 10^9^/L	12.8 (9.4–16.0)	11.3 (8.8–17.1)	.56
CRP, median (IQR), mg/L (n = 42)	84.6 (31.1–170.0)	67.4 (22.4–222.1)	.86

CRP values were available for 42 patients.

Abbreviations: BF-PP, BIOFIRE Pneumonia Panel; CRP, C-reactive protein; IQR, interquartile range.

^a^Data represent no. (%) of patients unless otherwise specified.

The median time (IQR) from admission to BF-PP test performance was similar between groups, 1 (0–2) days in the pathogen group and 1 (1–4) days in the nonpathogen group (*P* = .20). Overall, 77.1% of patients (84 of 109) had received antibiotic therapy before BF-PP testing (80.8% in the pathogen and 73.7% in the nonpathogen group; *P* = .38). Antibiotics had been initiated <24 hours before testing in 64.3% of patients in the pathogen group, compared with 50.0% in the nonpathogen group (*P* = .19). The antibiotics most commonly used before specimen collection were cephalosporins (81%), mainly ceftriaxone (55%), combined with either doxycycline or azithromycin. β-Lactam/β-lactamase inhibitor combinations and vancomycin were administered to 25% and 39% of patients, respectively. No significant differences in antibiotic exposure were observed between the pathogen and nonpathogen groups. Following BF-PP results, ceftriaxone remained the most frequently prescribed agent, either alone or in combination with doxycycline or azithromycin (Table 2).

**Table 2.** Antibiotic Treatment Before and After BIOFIRE Pneumonia Panel Results in All Study Patients

Antibiotic	Antibiotic Treatment, No. (%)	
	Before BF-PP (n = 84)	After BF-PP (n = 103)
Ceftriaxone	46 (55)	63 (61)
Cefepime	21 (25)	12 (12)
Cefazoline	1 (1)	0
Piperacillin-tazobactam	19 (23)	8 (8)
Amoxicillin-clavulanate	2 (2)	3 (3)
Doxycycline	31 (37)	21 (20)
Azithromycin	18 (21)	13 (13)
Vancomycin	33 (39)	15 (15)
Carbapenem	3 (4)	4 (4)
Levofloxacin	3 (4)	4 (4)
Other antibiotics	12 (14)	15 (15)

Abbreviation: BF-PP, BIOFIRE Pneumonia Panel.

Most patients were ultimately diagnosed with pneumonia, with a higher prevalence of pneumonia cases in the pathogen group (82.7%) than in the nonpathogen group (68.4%) (*P* = .08). CAP was more frequently observed in the pathogen than in the nonpathogen group (61.5*%* vs 43.9%, respectively; *P* = .06). Intubation rates during hospitalization were similar between groups (51.9*%* vs 50.9%; *P* = .91). The in-hospital mortality rate during the index admission was 21.2% in the pathogen and 14.0% in the nonpathogen group (*P* = .33). The median leukocyte count (IQR) was 12.8 × 10⁹/L (9.4–16.0 × 10⁹/L) in the pathogen and 11.3 × 10⁹/L (8.8–17.1 × 10⁹/L) in the nonpathogen group (*P* = .56), with similar neutrophil counts in both groups. The median C-reactive protein level (IQR) was higher in the pathogen group, at 84.6 (31.1–170.0) mg/L, compared with 67.4 (22.4–222.1) mg/L in the nonpathogen group, but the difference was not statistically significant (*P* = .86).

Table 3 presents demographics and clinical outcomes for the subgroup of patients assessed by ID specialists. The median age (IQR) was 59 (33–72) years in the pathogen and 64 (51–67) years in the nonpathogen group (*P* = .62). Most patients in both groups were male (81.8% vs 64.0%, respectively; *P* = .44). Common comorbid conditions across both groups were cardiovascular, GI, pulmonary, and neurologic diseases. The prevalence of cancer was higher in the pathogen group (45.5%) than in the nonpathogen group (28.0%) (*P* = .45).

**Table 3.** Baseline Characteristics and Clinical Outcomes in the *Streptococcus agalactiae* Pathogen and Nonpathogen Groups According to Infectious Diseases Clinician Assessments

Characteristic or Outcome	Patients, No. (%)^a^		*P* Value
	Pathogen Group (n = 11)	Nonpathogen Group (n = 25)	
Age, median (IQR), y	59 (33–72)	64 (51–67)	.62
Male sex	9 (81.8)	16 (64.0)	.44
Pulmonary disease	6 (54.5)	14 (56.0)	>.99
Gastrointestinal disease	8 (72.7)	17 (68.0)	>.99
Neurologic disease	6 (54.5)	14 (56.0)	>.99
Cardiovascular disease	7 (63.6)	23 (92.0)	.06
Renal disease	2 (18.2)	8 (32.0)	.69
Diabetes	4 (36.4)	9 (36.0)	>.99
Obesity	4 (36.4)	18 (72.0)	.07
Cancer	5 (45.5)	7 (28.0)	.45
Smoking	2 (18.2)	7 (28.0)	.69
Substance use (including alcohol)	1 (9.1)	8 (32.0)	.22
Time from admission to BF-PP testing, median (IQR), d	1 (1–5)	1 (1–2)	.38
Intubated during hospitalization	7 (63.6)	12 (48.0)	.39
In-hospital mortality	3 (27.3)	4 (16.0)	.65
Pneumonia	11 (100)	19 (76.0)	.15
Community-acquired pneumonia	4 (36.4)	17 (68.0)	.14
Prior antibiotic therapy	11 (100)	19 (76.0)	.15
Time before antibiotic initiation			
<24 h	7 (63.6)	8 (42.1)	.26
24–48 h	2 (18.2)	4 (21.1)	>.99
>48 h	2 (18.2)	7 (36.8)	.42
Leukocyte count, median (IQR), 10^9^/L	12.3 (5.5–15.8)	15.4 (9.8–17.8)	.06
CRP, median (IQR), mg/L (n = 17)	127.7 (55.8–159.2)	58.2 (33.2–233.2)	.70

CRP values were available for 17 patients.

Abbreviations: BF-PP, BIOFIRE Pneumonia Panel; CRP, C-reactive protein; IQR, interquartile range.

^a^Data represent no. (%) of patients unless otherwise specified.

All 11 patients (100%) in the pathogen group received antibiotic treatment before the BF-PP test, compared with 76.0% in the nonpathogen group (*P* = .15). Pneumonia was diagnosed in all patients (100%) in the pathogen group and 76.0% of those in the nonpathogen group (*P* = .15). Among these cases, CAP was identified in 36.4% in the pathogen and 68.0% in the nonpathogen group (*P* = .14). The rate of intubation during hospitalization was 63.6% in the pathogen group and 48.0% in the nonpathogen group, and the respective in-hospital mortality rates were 27.3% and 16.0% (*P* = .65).

### Microbiological Data

**Figure 2.**
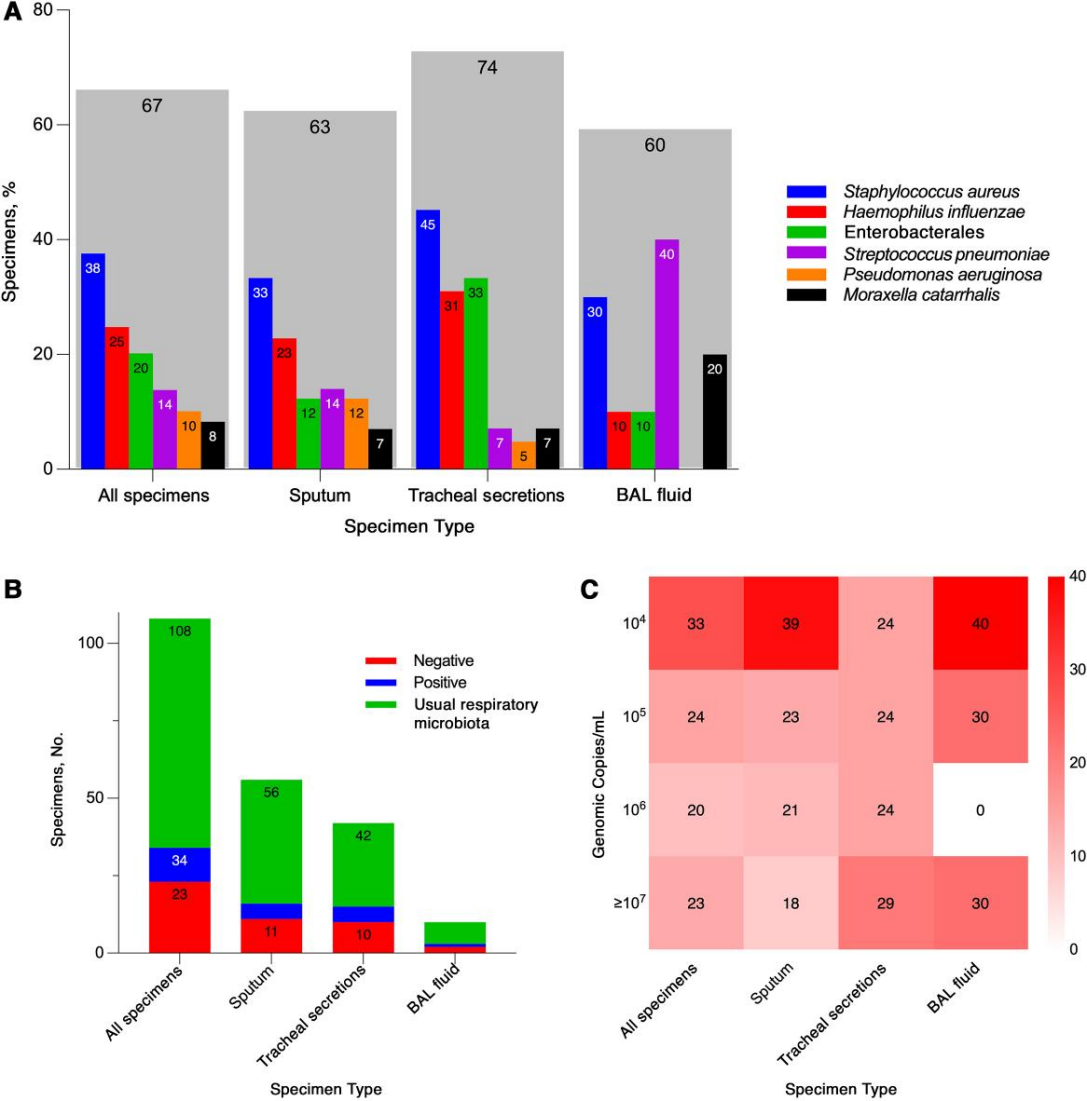
Microbiological findings by BIOFIRE Pneumonia Panel in *Streptococcus agalactiae–*positive respiratory specimens. *A*, Bacterial codetections by specimen type. Gray bars represent the overall proportion of specimens with any bacterial codetection, with individual bacterial detections shown as narrower bars within the same category. *B*, Culture positivity for *S agalactiae* by specimen type. Bars show the proportion of specimens reported as positive, negative or with usual respiratory microbiota. *C*, Semiquantitative distribution of *S agalactiae* genomic abundance across specimen types. Heat map values represent percentages of specimens within each genomic abundance category. Abbreviation: BAL, bronchoalveolar lavage.

GBS was detected by the BF-PP assay alone in 23.9% of specimens (26 of 109). In the remaining 83 specimens (76.1%), GBS was detected with other microorganisms, including bacteria and viruses. Figure 2 shows concomitantly detected bacteria, BF-PP semiquantitative GBS results, and concordance results with culture across all specimens, stratified by specimen type. Concomitant bacteria were detected in 67.0% of specimens, with the highest detection rate in tracheal secretions (74.0%), followed by sputum (63.0%) and BAL fluid (60.0%) specimens. The bacteria detected, in descending order of frequency, were *S aureus* in 41 specimens, *Haemophilus influenzae* in 27, Enterobacterales in 22, *Streptococcus pneumoniae* in 15, *Pseudomonas aeruginosa* in 11, and *Moraxella catarrhalis* in 9. The concordance between BF-PP results and culture was 10.2%, with similar rates across specimen types, and 68.5% of cultures were reported as containing usual respiratory microbiota. Semiquantitative BF-PP results for GBS across all specimen types were 10^4^, 10^5^, 10^6^ and ≥10^7^ genomic copies/mL in 33.0%, 24.0%, 20.0%, and 23.0% of specimens, respectively.

Viruses were codetected with GBS by the BF-PP in 24.8% of specimens (27 of 109), with influenza A detected in 10, human rhinovirus/enterovirus in 8, human metapneumovirus and parainfluenza virus in 3 each, RSV in 2, and coronavirus (not severe acute coronavirus disease coronavirus 2) in 1. Of the viral detections, 88.9% (24 of 27) were pneumonia-associated specimens, including 19 associated with CAP, 3 with HCAP, and 1 each with HAP and VAP.

## DISCUSSION

This study evaluated clinician interpretation (ID specialists and non-ID clinicians) of positive GBS BF-PP results and compared clinical outcomes between patients in whom GBS was considered a pathogen and those in whom it was considered a nonpathogen. GBS can colonize the GI and genitourinary tracts and, to a lesser extent, the oropharynx. Colonization rates reach 30%–47% in adults, with pharyngeal colonization reported to be between 4% and 12% [6, 19, 22]. Although GBS is an uncommon cause of pneumonia, it may play a pathogenic role in specific populations, particularly neonates and older adults.

The prevalence of GBS detection in patients with suspected pneumonia is higher when using molecular methods compared with standard culture. In a study of patients with pneumonia, GBS was detected in 5% by BF-PP, whereas culture identified only 0.4% [20]. While several studies have compared GBS detection by molecular assays versus culture in populations such as pregnant women, showing higher detection by the former [23, 24], there is lack of information evaluating this in those with suspected pneumonia.

Data on GBS interpretation in respiratory specimens are limited, which is why addressing this question was a central aim of the study. GBS was interpreted as a pathogen in approximately 48% of cases, while in the remainder it was considered either a colonizer or of unclear clinical significance. Notably, ID specialists classified GBS as a pathogen less frequently, in 30% of cases. Even when it was the sole microorganism detected, ID specialists rarely deemed it pathogenic, in contrast to non-ID clinicians. This suggests that clinical expertise influences interpretation, even when objective test results are provided.

Variability in interpreting diagnostic assays including BF-PP results has been well documented and carries implications for antimicrobial stewardship. Misclassification may lead to inappropriate antibiotic use, potentially undermining the utility of the panel. Previous studies on BF-PP implementation have reported mixed results; while some show no change in antibiotic use [25], others report reduced antibiotic administration duration in specific subgroups, such as those with viral coinfection [26], and the randomized controlled trial on which some data presented here is based [20], showed faster antibiotic escalations for both Gram-negative or Gram-positive bacteria, and faster antibiotc de-escalations for Gram-positive bacteria with the BF-PP implemented with antimicrobial stewardship. This variability extends beyond test interpretation. Although most patients in the pathogen and nonpathogen group were assessed by non-ID clinicians as having pneumonia, only 83% in the pathogen group were ultimately classified as such, despite GBS being identified as the causative agent, which is paradoxical. In contrast, ID specialists diagnosed pneumonia in 100% of these patients. This discrepancy likely reflects interclinician variability not only in test result interpretation (as previously highlighted) but also in clinical diagnosis of pneumonia, particularly in older adults, in whom presentation can be nonspecific; diagnostic uncertainty of pneumonia and inconsistency among clinicians have been previously reported [27]. Discrepancies were also observed in pneumonia type. Most pneumonia cases in patients assessed by non-ID clinicians were community acquired (62%), whereas in patients assessed by ID specialists, most cases were nosocomial (64%). Prior studies have reported similarly inconsistent patterns, although these were based on small cohorts and outdated data [28, 29].

Most patients in both groups were older adults, consistent with populations at higher risk for invasive GBS infection [15]. Clinical comorbid conditions did not differ significantly between groups, suggesting that comorbid conditions alone did not influence clinician interpretation. However, pulmonary diseases, although not significantly different between groups, were more frequent in the pathogen group. This trend suggests a potential predisposition to GBS pneumonia in patients with underlying pulmonary conditions, as chronic lung disease might increase susceptibility to respiratory infections by compromising mucosal defenses and altering local immunity [30].

The correlation between GBS detection and clinical outcomes remains debatable. In a study of patients with cystic fibrosis, GBS was found to be transiently detected and not associated with worse outcomes [31], while others report GBS as a cause of severe pneumonia [32]. A large US Veterans Health Administration study found that pneumonia accounted for 18% of deaths among patients with invasive GBS infection, with an overall 30-day mortality rate of 9% [15]. In the present study, the in-hospital mortality rate was higher in the pathogen than in the nonpathogen group (21% vs 14%). These findings suggest that classifying GBS as a pathogen may be associated with worse clinical outcomes; however, the small sample size limits definitive conclusions.

In addition to clinical features, it could be assumed that certain testing characteristics may influence the interpretation of GBS as a pathogen, such as detection in deep respiratory specimens, high genomic abundance, and detection of GBS as the sole microorganism. BAL fluids were more common in the pathogen group than in the nonpathogen group (13% vs 5%), with no significant difference in more superficial specimens. Although invasively collected specimens like BAL fluid are often perceived as more indicative of true infection, this alone did not reliably distinguish between groups.

In most cases, GBS was detected alongside other microorganisms, most commonly *S aureus* (38%). This aligns with studies reporting *S aureus* as a copathogen with GBS, though at low rates in pneumonia (7%) [15]. The higher rates of codetection observed here may reflect the enhanced sensitivity of molecular diagnostics compared to conventional cultures. In addition, the study period overlapped with the coronavirus disease 2019 pandemic, and *S aureus* is a common postviral bacterial pneumonia pathogen [33]. Codetection of GBS with other bacteria has been reported in other contexts. For example, GBS is frequently codetected with *P aeruginosa* in patients with cystic fibrosis [31] and *S aureus* in infants with nasopharyngeal carriage [34].

Reported concordance between BF-PP and culture for GBS detection varies (from 17% to 43%) [18, 35]. This appears to be influenced by genomic abundance level, reaching 77% at high levels (10^7^ copies/mL) and dropping to 9% at low levels [36]. In the current study, overall concordance was low (10%), however, approximately 70% of cultures reported “usual respiratory microbiota” without specifying bacterial species, possibly including GBS below workup or reporting thresholds. In addition, prior antibiotic exposure likely contributed to this discordance, as most patients had received antibiotics before specimen collection, possibly affecting culture positivity.

This study has several limitations. As a retrospective study, it is subject to missing and incomplete data. Some patients lacked documentation of follow-up of BF-PP results or did not provide permission to access their medical records and were therefore excluded from analysis. Classification of GBS as a pathogen, colonizer, or of unclear clinical significance was based on retrospective clinical interpretation, which is inherently subjective and may have introduced classification bias. As a single-center study, its findings may not be generalizable to other settings or populations with different demographic or clinical characteristics. The relatively small sample size also limited the statistical power of the study and precluded multivariable analysis. Finally, the association between interpretation of BF-PP results (particularly in cases of monomicrobial detection) and antibiotic decision making was not assessed. Further studies are needed to better define the clinical significance of GBS detection with the BF-PP.

In conclusion, the findings presented suggest that GBS is not an uncommon pathogen of pneumonia. However, it can also be detected as a nonpathogen, and there is considerable variability among clinicians in the interpretation of GBS-positive BF-PP results. No significant clinical differences were observed between patients in whom GBS was classified as a pathogen versus nonpathogen. Overall, its clinical significance remained unclear in more than half of cases. These findings underscore challenges interpreting GBS detection in respiratory specimens. Currently, there are no standardized criteria to distinguish colonization from true infection for microorganisms detected by the BF-PP, including GBS. The findings presented suggest that several factors may be relevant in developing such criteria, including clinical context, specimen type, codetections, and disease severity. Prospective studies evaluating clinician interpretation in real time are needed to validate these parameters. Such evidence could inform objective criteria, support antimicrobial stewardship efforts, and guide clinical decision making.
